# Zero Refrigerator

**Published:** 1869-09

**Authors:** 


ZERO REFRIGERATOR.—Hundreds of our best citizens, and thousands of families throughout the country consider this indispensable family convenience the coolest, cleanest and sweetest refrigerator yet devised, hence it is rapidly superseding all others. It is so contrived that, while it keeps provisions of every description uniformally cool in summer, it prevents them from freezing in winter. It does not acquire a closet smell because it is lined with zinc and all the shelving is metallic. The ice is put in at the top and the provisions through an opening in front, so that the two never come in contact. Dampness is impossible where the provisions are; hence they are always kept sweet.

The ice being uppermost and the articles to be preserved under it and at either side, the air is always cool and dry. The ice is kept in a close box, and not being exposed to a circulation of air, a small amount keeps a low temperature ; besides, the arrangement is such that cool drinking water is always at hand. The whole is filled with powdered charcoal, which is essential to the cleanliness of this ornamental piece of furniture, which has repeatedly taken the highest premium at various State fairs.

GOTHIC FURNACE for burning coal and wood is economical, powerful and durable. The prices range from $100 to $150. Numerous testimonials have been forwarded from various States as to the superiority of the Furnace, and as many persons are building houses into which they hope to move in the winter, it would be well to call and see Mr. LESLIE's stock before purchasing elsewhere, at 1310 Broadway, New York, or 605 Sixth Avenue.

MOUNT HOPE NURSERIES, Rochester, N. Y. (Established 1846.)—GENTLEMEN improving their grounds, ORCHARDISTS, LANDSCAPE-GARDENERS, NURSERYMEN, and DEALERS in Trees, will find our Stock of Fruit and Ornamental Trees the largest, and the collection the most extensive and complete in the U. S. All orders, large or small, will receive prompt and careful attention. Packing for distant points performed in the most skillful and thorough manner. Small parcels sent by mail when so desired. Descriptive and Illustrated priced Catalogues, sent pre-paid on receipt of stamps, as follows: No. 1.—Fruits, 10c. No. 2.—Ornamental Trees, 10c. No. 3.—Green house, 5c. No. 4.—Wholesale, FREE. Address—ELLWANGER & BARRY, Rochester, N. Y.

RUBY GRAY'S STRATEGY, by Mrs. Ann S. Stephens, is the title of an entirely new novel from the pen of this talented American authoress, now in press, and to be published in a few days by T. B. Peterson & Brothers, Philadelphia. The novels of Mrs. Stephens are productive of both pleasure and excitement. They are, moreover, always successful, for the reason, that while this gifted author is a conscientious follower of nature, she has also that fine artistic sense which teaches that nature, when shown within the lines of art, must be measurably heightened, colored, and enlarged. This is the real secret of successful writing—a secret appreciated by such masters of fiction as Dickens and Thackeray. There is no previous work of Mrs. Stephens so full of her peculiar power and genius—none so absorbing in conception and development as "Ruby Gray's Strategy." It is fully equal to her "Fashion and Famine." The price is only $1.75, bound in Cloth.

